# Ethical Dilemma: Should Continuous Intravenous Drug Use Affect Appropriate Management in Prosthetic Valve Endocarditis?

**DOI:** 10.7759/cureus.8458

**Published:** 2020-06-05

**Authors:** Talha Ahmed, Ayesha Safdar

**Affiliations:** 1 Internal Medicine, University of Maryland Medical Center, Baltimore, USA; 2 Internal Medicine, Army Medical College, Rawalpindi, PAK

**Keywords:** prosthetic valve endocarditis, infective endocarditis, native valve endocarditis, surgical valve replacement, intravenous drug use, continued drug use, indications for surgery

## Abstract

Drug use is a major challenge that negatively impacts many aspects of health. The issue of drug use is growing with every passing day. Efforts to mitigate its use are countered by even more people succumbing to the intravenous drug use due to their relatively easy availability and patients' poor insight into their medical condition. Infective endocarditis (IE) is a condition with high mortality and morbidity. It requires prolonged treatment with antibiotics, and, under some special circumstances, surgical management is also necessitated. Intravenous drug users who get valve replacement after index IE episode may continue to use drugs despite our utmost efforts to prevent it. They can subsequently develop prosthetic valve endocarditis (PVE), which is one of the indications for surgical valve replacement, hence requiring a redo surgery. However, their irregular behavior can create reservations while considering a repeat valvular surgery and delay the appropriate treatment. This can increase morbidity and mortality from PVE in intravenous drug users with otherwise no or few comorbidities.

## Introduction and background

According to a report by the National Institute of Drug Abuse (NIDA), the estimated cost burden of illicit drug use on healthcare is 11 billion dollars, whereas their overall cost burden was 193 billion dollars in the United States in 2007. NIDA predicted this number to be even higher for the report issued in 2010 [1]. One study reported that drug use-related infective endocarditis (IE) is on a rise geographically throughout the United States, with Midwest having the highest annual percentage increase. The results of the study showed a doubling of cases of drug use related IE from 2002 (8%) to 2016 (16%). This has been accompanied by an increase in the requirement for valvular surgeries required for these patients. Intravenous drug use (IVDU) is increasing among IE patients requiring surgery. This has been confirmed by the results of multiple studies across the United States [2].

With the opioid crisis affecting the United States in the last decade, an increase in admissions for IE has been observed. These patients with poor insight into their medical condition do not realize the root cause of their disease. Even after getting valve replacement surgery, the drug use behavior is continued in the majority of cases. There have been studies recently showing no added benefit of surgical valve replacement in these patients. This primarily was due to their ongoing risk of infection [3].

In resource-poor countries, this issue can prove to be costly and even fatal. In developed and resourceful countries, an ethical dilemma arises. When providers see the same patient coming back with IE of the prosthetic valve due to continued IVDU, reservations arise. The critical thinking that goes into this decision-making delays their appropriate treatment (surgical valve replacement). Even if it is agreed upon to perform surgery, the patient is exposed to risks of morbidity and mortality from delayed management, which could have been prevented [4]. This review will focus on this dilemma and measures that can be taken to address it.

## Review

Despite advancements in valve repair and replacement therapies, the treatment of prosthetic valve endocarditis (PVE) requires surgical replacement. The surgical procedure has its inherent risks. Medical management with antibiotics is the cornerstone of treatment for native valve endocarditis (NVE). Patients with PVE who most likely to benefit from this approach are those who remain clinically stable or show improvement on antibiotic treatment. High-risk echocardiographic features such as valvular abscess, valve dysfunction or dehiscence, and infection spreading beyond the leaflet of the bioprosthesis may warrant surgery. On the other hand, PVE caused by less virulent organisms such as coagulase-negative Staphylococcus and viridans Streptococcus species can be managed by medical management. For patients at high risk of surgery and also those who do not consent, medical management alone may be reasonable [5,6].

A consensus approach to the treatment of PVE in patients who continue IVDU is lacking. This is because of a lack of well-defined study including these participants. All retrospective studies are limited by a lack of randomization along with other conventional biases associated with retrospective studies. Results from one study showed that treating stable patients with PVE with antibiotics is a reasonable approach. However, they should be followed closely and, if not improving clinically or on echocardiography, should undergo surgical management. This approach, however, cannot be generalized as the disease is very unpredictable and carries a high mortality and morbidity. Also, in this group of patients (intravenous drug users), a close follow-up while desirable is often not possible [7,8].

In the recent 2015 American College of Cardiology/American Heart Association (ACC/AHA) guidelines as well as the European Society of Cardiology (ESC) guidelines, patients with NVE should be managed at reference centers by a specialized “endocarditis team”. This has been advocated to closely monitor a need for surgical valve replacement for these patients, and hence a cardiothoracic surgeon should be part of the team [9,10]. The current American Association for Thoracic Surgery (AATS) guidelines also emphasize this fact. Though there are minor differences, both the AATS and ACC/AHA guidelines currently recommend surgical valve replacement for PVE. However, they do not mention that active intravenous drug users are excluded from these recommendations. In the light of this evidence, an intravenous drug user has an equal right to valve replacement as a non-intravenous drug user [11].

A recent study reported by 2019 by Straw et al. analyzed patients with IVDU and IE and compare them with patients with IVDU who had other infections besides IE. In this interesting prospective observational study, the outcomes of interest were all-cause mortality, cause of death, relapse/recurrence, and re-operation. The results of this study showed that patients with IVDU and IE had worse primary outcomes. This was particularly true for that subset of patients with IVDU and IE who underwent surgical replacement (hazard ratio: 1.8; 95% CI: 0.95-3.3). The commonest cause of death in these patients was related to infections mostly being repeated episodes of IE (55%) [12]. The results of this study argue against the utility of undergoing valve replacement surgery in patients with continued IVDU who have NVE. With PVE and continued drug use, the utility of surgical replacement should be questioned even more based on the results of this analysis.

Another observational study by Østerdal et al. analyzed patients with continued IVDU and IE. A total of 52% of their patients presented with PVE, demonstrating a reinfection rate of 25-28%. In their study, 70% of patients continued with IVDU at the time of discharge after getting valve replacement surgery for index NVE [13]. The authors of the study also raised the ethical dilemma of a redo surgery and concluded that in severe cases of heart failure, cardiogenic shock, and profound sepsis, valve replacement is reasonable for PVE.

In an attempt to answer this question, Miljeteig et al. conducted an ethical analysis comprising seven key questions. The conclusions made in their study were based on the principle of equity [14]. This suggested that intravenous drug users should be treated the same as other patients based on the principle of equal treatment and expected survival. A second valve replacement surgery may provide these patients with an additional one to two years of life expectancy. However, if after risk-benefit analysis, surgery was not going to prolong life, it should be avoided. They also emphasized the importance of counseling these patients at all levels and following up closely. The majority of the authors were in favor of valve replacement surgery. The rest of the minority emphasized assessing patient's willingness to quit drug use through various means prior to valve replacement.

In some small medical centers that are not equipped with the cardiothoracic surgery team, it becomes even difficult to manage the PVE patients. These centers rely on the surgical services of larger tertiary care centers. The whole process of coordinating the admission of these patients with the surgical team based at another center makes the management challenging. These patients may not be prioritized as compared to if they were present in that tertiary care center itself. However, every effort should be made to transfer these patients, even if they are clinically stable, to a large tertiary care center in the premises of the small center. This ensures that a full-fledge endocarditis team is on-board to manage patients with PVE [15,16].

The clinical presentation of these patients might also play a role in decision-making. For patients who are in refractory shock (septic and cardiogenic combined), some form of mechanical circulatory support may be required. There have been case reports of valve replacement procedures including surgeries being performed under these supports [17,18]. Each invasive modality of treatment has some implications in the form of short- and long-term complications. In patients with PVE, this factor should also weigh in before considering the use of these devices. A well-developed risk prediction model taking into account the drug use behavior seems to be a rational approach in these patients. If those few years of life that surgery adds to these PVE patients’ life span are spent fighting with the complications of these invasive devices, then we should really ask ourselves the question of what good are we doing to these patients and to the society?

One of the ways of addressing this situation is to address the intravenous drug users’ behavior of drug use. After they get the first valve replacement surgery for NVE, an extensive discussion and counseling should be undertaken. A multi-disciplinary approach should be adopted at the index valve replacement visit to prevent re-admission for PVE. This should comprise of a dedicated addiction team, a possible psychiatric evaluation, and various forms of behavioral therapies that should be offered. A list of places to treat withdrawal from drugs should be given to these patients. If patients are willing, families should be involved and every possible attempt at recruiting social support and help for these patients should be considered before discharging them from the hospital.

Hull and Jadbabaie in their brief review undertook a very interesting discussion in an attempt to address this concern. They recommend a "three-strike approach". They recommended a policy where a physician’s obligation to treat all patients with equity should be balanced alongside patients’ behavioral management and attempt to quit drug use. They proposed that at index valve replacement, a full-blown effort to address drug use should be undertaken. Patients should be made to sign a contract stating they will make all possible efforts and cooperate with addressing their drug use [19]. However, for patients who fail to comply and present the second time, a second chance should be given to them but very clearly stating that this is their "last chance". This approach though rational can be challenged as the legality of the contract can be questioned by the patients and their families. Moreover, in patients who continue drug use and develop PVE, the “signed contract” can create an aversion for these patients to utilize healthcare.

Based on the aforementioned studies, a broad approach to the management of these patients is needed. This should start with outpatient visits. All intravenous drug users should be counseled regarding developing IE at every outpatient visit. A thorough effort should be undertaken to assess their comprehension and insight into their drug use behavior and the risk of developing IE. In addition, patients who develop other IVDU-related infections such as human immunodeficiency virus (HIV) and hepatitis C and require frequent follow-ups should be counseled during each of their follow-up regarding drug use and IE. Even after these preventive efforts, for patients who develop an index episode of IE and are intravenous drug users, a "multi-modal native valve endocarditis approach" should be a part of their treatment. Involving the hospital ethics committee, social worker, and transitional care team, psychiatric evaluation, and strengthening social support by recruiting family members and loved ones can improve their chances of trying to address their drug use [20,21]. Ideally, these patients should be discharged to facilities where their drug use can be addressed at the same time they are completing their course of antibiotic treatment. Through these measures, we can possibly mitigate the risk of PVE in these patients since drug use itself is a chronic disease that needs close monitoring and proper attention.

## Conclusions

The principle of equity implies offering standard-of-care treatment to all patients. In intravenous drug users who develop PVE from continuous drug use, this approach is reasonable in resource-rich countries. However, in resource-poor countries, this can be challenging, particularly when these patients develop reinfection within the first few years of their index surgical valve replacement. One can argue that even in developed countries, patients on list for valve replacement who are non-intravenous drug users are more than the availability of the product (prosthetic valves). The question worth asking is: is the quality of that one to two years of life that the second valve replacement offers to the PVE with continued IVDU gratifying, satisfactory, and worthwhile? There is no definitive answer to this ethically challenging question. However, a more prudent way to address this issue is a holistic multi-disciplinary approach to address the drug use at the first valve replacement procedure. It should be our standard of care. This will not only reduce the healthcare utilization of these patients by preventing the re-infection of prosthetic valves but will also help us preserve our limited resources for other deserving patients who are on the waiting list for these limited yet precious and life-saving resources.
